# Effect of Buckwheat Husk Addition on Antioxidant Activity, Phenolic Profile, Color, and Sensory Characteristics of Bread

**DOI:** 10.3390/molecules30173625

**Published:** 2025-09-05

**Authors:** Wajeeha Mumtaz, Marta Czarnowska-Kujawska, Joanna Klepacka

**Affiliations:** Department of Commodity Science and Food Analysis, The Faculty of Food Sciences, University of Warmia and Mazury in Olsztyn, 10-719 Olsztyn, Poland; marta.czarnowska@uwm.edu.pl (M.C.-K.); klepak@uwm.edu.pl (J.K.)

**Keywords:** buckwheat, by-product, bioactive compounds, phenolics, innovation in bread making, fortified breads, sensory evaluation

## Abstract

The incorporation of bioactive compounds from plant-based by-products into staple foods represents a sustainable strategy to enhance both nutritional quality and health benefits. The aim of this study was to evaluate the effect of buckwheat husk addition (1.5%, 3.0%, 4.5%) on the antioxidant activity, total phenolic content (TPC) and its profile, color parameters, and sensory attributes of wheat and wholemeal breads. Increasing the husk content significantly (*p* ≤ 0.05) enhanced antioxidant activity, especially in the lipid-soluble fraction, with the highest values observed at 4.5% addition. In terms of TPC, wheat bread showed a significant (*p* ≤ 0.05) increase (16.5%) only at 3.0% husk addition, while wholemeal breads exhibited consistent TPC growth at all levels, reaching a 35.2% increase at 4.5% enrichment. Phenolic profiling revealed syringic acid as the dominant compound, constituting up to 64.4% of total phenolic acids in wholemeal bread with 4.5% husk. Flavonoids content increased with husk addition, with rutin, catechin, and orientin most prominent. Color analysis indicated a reduction in lightness and hue angle, an increase in browning index and total color difference with higher husk addition. Addition of husk modified aroma, color, and mouthfeel. Wholemeal breads with 1.5% and 4.5% buckwheat husk had the highest acceptability, enhancing nutritional and functional quality without affecting preference. Buckwheat husk effectively enhances bread’s nutritional and functional quality.

## 1. Introduction

Healthy food products that enhance mental and physical health and prevent diseases linked to nutrition are of importance to consumers [1]. Bread and other food items could serve as vehicles for adding functional substances that have positive health effects for customers [2,3]. Regardless of its kind, method of manufacture, or place of origin, bread is often made from refined common wheat (*Triticum aestivum*) flour but also fortified with whole-grain wheat flour, flour made from gluten-free cereals (rice, maize, sorghum, millet), or flour from pseudocereals (quinoa, buckwheat, and amaranth) and traditional grains (wheat, rye, barley, oats, spelt, triticale) [4]. Bread enrichment has advanced due to better techniques and rising health, economic, and environmental awareness [5]. Bread has been enriched with dietary deficient ingredients in the synthetic form such as folic acid and fiber and minerals such as iron, calcium, magnesium, zinc, potassium, and phosphorus [6]. In addition to this trend, bread has been enriched with variety of natural plant-based ingredients such as chia, amaranth, quinoa, sorghum, almond flour, pea protein, cabbage powder, carrot powder, sweet potato powder, moringa powder, cabbage seeds, and lupin flour [7,8]. These plant-based additions are chosen for their nutritional benefits, including high fiber content, unsaturated fatty acids, and antioxidant properties, which contribute to the functional properties of the bread.

Particularly important are ingredients that have a high content of antioxidant compounds such as polyphenols [9]. Polyphenols are powerful antioxidants that help neutralize free radicals, reducing oxidative stress and lowering the risk of chronic diseases like cancer, cardiovascular issues, and neurodegenerative disorders. They also have anti-inflammatory effects and support heart, brain, and metabolic health [10]. Significant amounts of phenolic-rich byproducts are produced by the food and agricultural processing industries, which may be important natural sources of antioxidants when added to staple food like bread. Recent research has shown that a number of these by-products, including pistachio hulls, grape skin, peels from apples, peaches, pears, and citrus are a good source of phenolic antioxidants [11,12,13].

Due to their nutritional and antioxidant potential, these by-products are increasingly being used to enrich bread and other baked products. Among the various plant-based sources used to improve the nutritional quality of bread, pseudocereals have gained particular attention for their balanced nutrient content and bioactive compounds. Buckwheat, a nutrient-rich pseudocereal, has recently gained attention not only for its grains but also for its underutilized by-products, such as buckwheat husk, which show promising potential as functional food ingredients. Buckwheat husk, the outer protective covering of buckwheat seeds, are a significant by-product in regions where buckwheat is cultivated. Russia, China, and Ukraine are among the world’s leading producers of buckwheat, however, in Poland cultivation of this cereal is no less popular [14]. Buckwheat husk is a rich source of bioactive compounds, including phenolic acids and flavonoids, which contribute significantly to their antioxidant properties. These beneficial compounds are primarily concentrated in the outer layers of the buckwheat seed, making the hulls a valuable by-product in the processing of buckwheat seeds [15]. Buckwheat husk shows 2–5 times more phenolic compounds than whole buckwheat grains and 2–7 times higher antioxidant activity compared to other grains such as barley, triticale, and oats [16]. Buckwheat husk is also a rich source of flavonoids, including rutin, quercetin, and kaempferol. These compounds contribute to the husk antioxidant and anti-inflammatory effects [17].

In the available literature, there are only few studies in which buckwheat hulls alone were added to bread, because the most commonly used buckwheat additive is flour obtained from whole or dehulled buckwheat seeds, in which the starchy endosperm predominates. For instance, Wronkowska et al. (2019) evaluated the use of roasted buckwheat flour and hulls (raw and roasted) in wheat rolls and mixed rye-wheat breads, while Gutiérrez et al. (2023) reported improvements in dietary fiber and antioxidant capacity in gluten-free breads enriched with buckwheat hull particles; however, these studies were limited to specific product types. In contrast, the present study assesses the fortification of both wheat and wholemeal breads with varying levels of buckwheat husk. It offers new insights into the application of buckwheat husk in conventional bread production [18,19].

Due to the growing interest in innovative additives that not only appeal to consumers’ sensory preferences but also contribute to the nutritional improvement of staple foods, buckwheat husk was chosen as a functional ingredient for bread enrichment. The aim of the study was to evaluate how buckwheat husk addition affects parameters related to the antioxidant and sensory properties of bread enriched with buckwheat hulls. Test materials consisted of two types of wheat bread with the addition of commercially available grounded buckwheat husk.

## 2. Results and Discussion

### 2.1. Antioxidant Activity and Total Content of Phenolic Compounds

The antioxidant activity of bread samples with varying concentrations of buckwheat husk (0%, 1.5%, 3.0%, and 4.5%) was assessed through the measurement of both water-soluble antioxidant compounds (ACW) and lipid-soluble antioxidant compounds (ACL), as well as their total antioxidant activity (PCL) expressed in µmol Trolox equivalents per gram of dry mass (dm). The results obtained for bread samples enriched with buckwheat husk showed significant (*p* ≤ 0.05) variations in antioxidant activity between different treatments (type of bread, buckwheat husk concentration), with a clear trend of increased antioxidant activity as the concentration of buckwheat husk increased (Table 1). Only the wheat bread (WB) sample with 1.5% buckwheat husk (WB 1.5%) addition exhibited lower water-soluble antioxidant activity (0.79 µmol Trolox/g) than the control sample WB0 (0.87 µmol Trolox/g) (Table 1). In the other enriched samples of bread, a significant (*p* ≤ 0.05) increase in antioxidant activity measured with ACW protocol, varying from 8.5% (WMB 1.5%) to nearly 50% (WB 4.5%) was observed. This marked increase showed that the addition of buckwheat husk contributes to a higher concentration of water-soluble antioxidant compounds in the bread matrix. 

A similar pattern was observed for lipid-soluble antioxidants. The higher the addition of the husk, the higher the value of antioxidant activity measured with the ACL protocol, with the highest value for wholemeal bread with 4.5% buckwheat husk (3.91 µmol Trolox/g) (Table 1). The resulting increases in antioxidant activity measured by ACL, varying from 16.8% (WMB 1.5%) to 362.7% (WB 4.5%), were much higher than the observed increases in ACW at the same levels of husk enrichment of the bread. This significant (*p* ≤ 0.05) increase in antioxidant activity in the lipid-soluble fraction further supports the hypothesis that buckwheat husk is a valuable source of antioxidant compounds, which are more soluble in organic solvents such as lipids than in water. Antioxidants in buckwheat were more effectively extracted with organic solvents like methanol and acetone than water [20]. Moreover, a much higher increase in ACL compared to ACW in wheat bread samples than in wholemeal bread samples was observed. ACL increased more in wheat bread than ACW, likely due to the better extractability of fat-soluble antioxidants such as tocopherols and carotenoids in the simpler wheat white bread matrix, whereas these compounds may be bound or less accessible in wholemeal bread [21]. Although wholemeal bread had higher antioxidant levels initially, the structure of wheat bread allows for faster diffusion and measurement of added lipophilic antioxidants [22]. In wholemeal bread, the higher fiber content can interfere with mixed micelle formation by entrapping lipids and bile salts, which reduces the solubilization and extractability of lipophilic antioxidants. This sequestration, along with increased viscosity, limits diffusion and extractability, so ACL increases are smaller, despite higher overall antioxidant levels. Conversely, the simpler, lower fiber matrix of wheat bread allows lipophilic antioxidants to be more readily released and measured [23].

While our study focused on lipophilic antioxidant activity in a baked matrix, related work on fermented buckwheat showed significant enhancement in antioxidant capacity (e.g., DPPH, ABTS) and phenolic release, influenced by enzymatic activity and solvent polarity. These findings support the broader impact of processing on antioxidant extractability in buckwheat-based systems [24]. Although cold plasma is a non-thermal method, it has been shown to significantly enhance the antioxidant activity and phenolic bioaccessibility of fruit-based systems. This improvement was attributed to CP-induced cell wall disruption, which facilitated the release and solubility of phenolic compounds and vitamin C without compromising sensory quality. Such findings align with our results, where matrix modification during baking improved the extractability of antioxidant compounds [25]. In a related study, soybeans subjected to solid-state fermentation with *Eurotium cristatum* YL-1 exhibited a marked increase in total phenolics, antioxidant capacity, and isoflavone aglycones. These enhancements were strongly correlated with enzymatic activity, particularly β-glucosidase, highlighting how bioprocessing improves the functional properties of plant matrices [26].

The total PCL, which was calculated as a sum of results from ACW and ACL measurements, was the lowest (1.46 µmol Trolox/g) in wheat bread without buckwheat husk added (WB 0). Nonenriched wholemeal bread (WMB 0) showed more than 2.5 times higher PCL (3.84 µmol Trolox/g) (Table 1) compared to WB 0. Whole-grain flour is made from the whole wheat kernel, including the bran, germ and endosperm, which preserves more bioactive compounds such as phenolic acids, flavonoids and fiber. These compounds, which are known for their antioxidant properties, significantly contribute to the higher antioxidant capacity in wholemeal bread (WMB). In contrast, wheat bread (WB) is made from refined wheat flour, which undergoes milling that removes the bran and germ, the parts of the grain richest in phenolic compounds. As a result, wheat bread (WB) contains less compounds with antioxidant potential compared to wholemeal bread. Therefore, the inclusion of wholemeal flour for bread preparation is more nutritionally beneficial [27]. In our study, the effect of buckwheat husk addition on antioxidant activity measured with PCL was higher in wheat bread samples with a PCL increase of 49.3%, 123.3%, 174.7% in WB 1.5%, WB 3.0%, and WB 4.5%, respectively, due to the initial lower antioxidant potential of wheat bread in comparison to wholemeal bread samples. The total phenolic contents for the control samples were 110.5 mg/g for wheat bread (WB 0) and 97.0 mg/g for wholemeal bread (WMB 0) (Table 1).

Interestingly, the solvent-extractable TPC of the wheat bread control (WB 0) was higher than that of the wholemeal bread control (WMB 0) (Table 1). This apparent contradiction can be explained by differences in the form and extractability of phenolic compounds. In cereal grains, the vast majority (≈80–95%) of phenolics are covalently linked (through ester or ether bonds) to cell-wall polysaccharides such as arabinoxylans or lignin, while only a minor fraction occur as free or soluble conjugated forms; for example, ferulic acid exists in free–conjugated–bound ratios of about 0.1:1:100 [28]. These insoluble, bound phenolics are often called “unextractable phenolics” because they are not released by aqueous or aqueous-organic solvents and require acidic, alkaline, or enzymatic hydrolysis [29]. Consequently, analytical procedures based solely on solvent extraction (e.g., the Folin–Ciocalteu assay) mainly quantify the free phenolic fraction and can underestimate total phenolics in whole-grain matrices [30]. Refined wheat flour contains fewer total phenolics, but those present are predominantly in free or soluble forms and are more readily extracted and detected. Conversely, wholemeal bread is richer in bound phenolics associated with the bran and fiber network, which remain largely unreleased under the extraction conditions used, resulting in a lower measured TPC [31,32,33]. Moreover, during mixing and baking, some bound phenolic complexes in refined dough may be disrupted, and Maillard reaction products formed from phenolics and proteins can contribute to Folin–Ciocalteu absorbance [34]. Taken together, these factors explain why the measured TPC of the wheat bread control was higher than that of the wholemeal bread control, even though wholemeal products contain more total phenolics in bound form.

In wheat bread (Table 1), only the addition of 3.0% buckwheat husk (WB 3.0%) caused a significant (*p* ≤ 0.05) TPC increase (16.5%) when compared to the control sample (WB 0). Contrastingly, in wholemeal bread, all tested levels of buckwheat husk addition resulted in a significant (*p* ≤ 0.05) increase in TPC of 15.2% (WMB 1.5%), 31.7% (WMB 3.0%), and 35.2% (WMB 4.5%). In the wholemeal bread (WMB) samples, the obtained results for total phenolic content are consistent with the results obtained for antioxidant activity, as both showed a significant increase with higher buckwheat husk additions. The inconsistent trend in TPC observed in wheat bread enriched with buckwheat husk may be related to the presence of antioxidants such as tocopherols and carotenoids (as discussed above), but may also result from interactions between phenolic compounds and wheat flour proteins, primarily gluten-forming proteins such as gliadin and glutenin [35]. Recent studies suggest that polymeric phenolics, such as tannins, possess flexible structures with multiple hydroxyl groups, which enhance their capacity to form dense networks with gluten proteins. This structural feature may contribute to stronger gluten-phenolic complexes, particularly in refined wheat bread, affecting phenolic behavior during processing [36]. Polyphenols can bind to proteins via non-covalent interactions, including hydrophobic bonding and hydrogen bridges, which reduces their solubility and extractability. At low husk levels, the introduced phenolics may have been tightly bound by the protein matrix, masking their detection. On the other hand, at high husk levels, excess fiber may have further impeded extraction efficiency or limited phenolic release during baking. These protein polyphenol interactions are particularly important in white wheat bread, where the absence of bran reduces the natural buffering capacity and phenolic diversity seen in wholemeal formulations [37]. This can explain the trend observed in our study that TPC only increased significantly at 3.0%, when the balance between available phenolics and matrix binding might have favored better extraction.

This study clearly indicates the potential of the buckwheat husk as an additive to increase the antioxidant potential of bread. These results are consistent with studies showing that the antioxidant potential of plant derived materials, such as buckwheat husks, can be effectively transferred into valuable food products during processing. Shyu et al. (2013) showed citrus peel powder, a fruit processing by-product, was added to toast bread and significantly improved antioxidant activity and sensory acceptability, especially at 4–6% levels of unfermented powder dried at high temperatures [38]. Another author evaluated the positive impact of adding pomegranate peel powder to pan bread on its nutritional quality and shelf-life characteristics. Compared to the control, bread enriched with 1% pomegranate peel powder showed increased fiber content, improved staling resistance, and maintained the highest sensory acceptability among the tested levels (1%, 2%, and 5%) [39]. In another study, watermelon rind powder (WMRP) added to wheat flour significantly enhanced the antioxidant properties of pan bread. As the level of WMRP increased, the total phenolic content and antioxidant activity, measured by DPPH radical scavenging, were significantly higher compared to the control bread. Specifically, the bread with 6%, 9%, and 12% WMRP showed a marked increase in antioxidant activity, with the highest levels of substitution resulting in the most notable improvements [40]. In a similar study, colored rice bran was found to contain up to 97% of phenolic acids in bound form, requiring special extraction methods to release them. In contrast, buckwheat husk used in this study released phenolics effectively during baking, confirming its functional advantage as a readily available antioxidant source [41].

### 2.2. HPLC Profile of Phenolic Compounds

In the tested wheat and wholemeal bread samples enriched with various levels of buckwheat husk (1.5%, 3.0%, and 4.5%) thirteen phenolic acids were identified in all types of wheat and wholemeal bread samples, regardless of the level of buckwheat husk addition. The content of phenolic acids and flavonoids in wheat and wholemeal breads with different levels of buckwheat husk is summarized in Table 2. The total content of phenolic acids significantly (*p* ≤ 0.05) increased in wheat bread (WB) samples with 3.0% and 4.5% buckwheat husk addition when compared to the control sample (WB 0) and in wholemeal bread (WMB) with 1.5% buckwheat husk, compared to its respective control sample (WMB 0). Ambiguous changes in the sum of phenolic acids occurring under the influence of adding buckwheat husk in both types of bread may result from different forms of interactions of these compounds with fiber. On the one hand, 1.5% addition of husk to wheat bread causes a decrease in the sum of phenolic acids because they bind to the fiber present in this amount of husk, while adding it at a higher level, in addition to fiber, also introduces a higher, non-fiber-bound content of phenolic acids, which increases their analytically detectable content. On the other hand, whole-grain bread contains a higher content of phenolic acids and fiber than wheat bread, therefore the addition of buckwheat husk, rich in both fiber and phenolic acids, influences a slightly different direction of their mutual interactions. Gua and Beta (2013) stated that phenolic acids can bind to both soluble and insoluble fiber, and the degree of these interactions depends on their proportions and origin. Zheng et al. (2024) added that depending on the raw materials from which these compounds originate, they can form various types of chemical bonds, both covalent and non-covalent, which affects the level of phenolic acids detected [31,32]. Syringic acid was the predominant phenolic acid identified in almost all tested bread samples. It was found that the incorporation of buckwheat husk at the highest enrichment level significantly (*p* ≤ 0.05) influenced the level of syringic acid in both wheat and wholemeal bread. The content of syringic acid in WMB 4.5% buckwheat husk was at the level of 4.275 µg/g and 3.213 µg/g in WB 4.5%, which represented, respectively, 64.4% and 49.4% of total phenolic acids. The increase in syringic acid content with higher levels of buckwheat husk addition is attributed to the thermal release of bound phenolic compounds during baking. Buckwheat husk contains phenolic acids such as syringic acid predominantly in bound forms, linked to cell wall components (such as lignin, hemicellulose, and cellulose) through ester bonds. During heat processing, these bonds are cleaved by heat, disrupting the plant matrix and facilitating the release of syringic acid into its free, extractable form [42]. Similar results were reported by Różańska et al. [43], who observed a significant increase in syringic acid content from 2.12 µg/g to 7.77 µg/g in the crumb and from 2.25 µg/g to 8.41 µg/g in the crust of roasted buckwheat bread, attributing this to heat induced transformation of phenolic compounds during processing. Vanilic acid showed a clear increase after buckwheat husk addition, confirming the potential of buckwheat husk to improve the phenolic acid profile of bread. Ferulic acid was also identified among the phenolic acids, with the highest concentration observed in WMB 1.5% at a level of 5.981 µg/g. It was found that the incorporation of buckwheat husk at the 1.5% enrichment level significantly (*p* ≤ 0.05) influenced the formation of ferulic acid in wholemeal bread. Ferulic acid is commonly present in cereal matrices in bound form, and its release during baking may be associated with the breakdown of cell wall structural components such as arabinoxylans and lignin. However, in WMB 1.5%, the concentration of ferulic acid (5.981 µg/g) exceeded that of syringic acid (2.534 µg/g), indicating that both the level of husk addition and the bread matrix can influence the distribution of individual phenolic acids. Ferulic acid is recognized for its potent antioxidant properties and likely contributed to the antioxidant activity observed in the enriched bread samples [44].

Eleven flavonoids were identified in tested breads samples. The sum of flavonoids increased significantly (*p* ≤ 0.05) with higher levels of buckwheat husk addition in both wheat and wholemeal bread. In wheat bread (WB), the highest flavonoid content was 0.33 µg/g at 4.5% buckwheat husk addition, while in wholemeal bread (WMB) it reached 0.39 µg/g at the same enrichment level. Catechin, myricetin, orientin, and vitexin contents were calculated in all eight bread samples (both wheat and wholemeal) although their levels were very low and varied significantly (*p* ≤ 0.05) among samples. Kaempferol was found only in wheat bread (WB) samples. Rutin was detected only in bread samples with added husk, with a statistically significant (*p* ≤ 0.05) increase observed in wheat bread. As there are no direct studies on the flavonoid profile of bread enriched with buckwheat husk, our results are novel and show similarities with previous studies on plant-based ingredients [38,39]. Kaempferol and myricetin are unevenly distributed in plants and often occur in very small amounts or are absent, depending on the plant type and processing [45]. These findings support our results and suggest that the presence of specific flavonoids, especially kaempferol, may depend on the type of flour used and interactions during baking. Another study reported that rutin appeared only in bread enriched with wholegrain buckwheat flour and increased with its higher additions, which is consistent with our results where rutin was detected only in husk enriched bread samples [46]. With regards to rutin, its content decreased significantly above 150 °C, showing its sensitivity to heat, which explains the low rutin levels in our bread samples [47]. Rutin content decreased during dough preparation and baking, mainly due to enzymatic hydrolysis and thermal degradation. During dough preparation, about 85% of rutin in Tartary buckwheat flour was hydrolyzed into quercetin due to the activity of endogenous enzymes activated by the addition of water and yeast. After baking, rutin was detectable only in bread made with 100% Tartary buckwheat flour, indicating its low stability during the bread-making process, as reported by Vogrincic et al. [48]. Based on our results for bread enriched with buckwheat husk, it can be concluded that phenolic acids, rather than flavonoids, were the primary contributors to total polyphenol content. 

### 2.3. Assessment of Color Changes

The evaluation of appearance characteristics showed noticeable trends across the different color parameters (Table 3). In wheat bread, the browning index (BI) increased significantly (*p* ≤ 0.05) from 2.98 in the control (WB 0) to 5.26 at 4.5% buckwheat husk addition (WB 4.5%). Also, in wholemeal bread (WMB), a similar significant increase (*p* ≤ 0.05) was observed, with the BI reaching 6.78 at 4.5% enrichment (WMB 4.5%) compared to the control (5.74, WMB 0). These results showed that the incorporation of buckwheat husk enhances browning, particularly at the highest level of enrichment. In wheat bread (WB), lightness (L) decreased from 75.31 in the control sample (WB 0) to 59.00 at 4.5% buckwheat husk addition (WB 4.5%), indicating a significant darkening of the bread crumb. In wholemeal bread (WMB), the lightness declined significantly (*p* ≤ 0.05) from 73.81 (WMB 0) to 54.32 at 4.5% buckwheat husk addition (WMB 4.5%). This progressive darkening reflects the influence of the natural pigments present in buckwheat husk.

Buckwheat husk addition resulted in a significant (*p* ≤ 0.05) color saturation decrease in all tested samples apart from WMB 4.5%. Wheat bread husk addition resulted in a significant (*p* ≤ 0.05) decrease in hue angle level, indicating a perceptible shift toward a duller and less yellow crumb tone. Such marked changes in h° were not evident in enriched wholemeal bread samples. With increased husk addition, the increase in total color difference was observed in all tested bread samples. Similar observations were reported by Gutiérrez et al. [19], who found that the addition of buckwheat husk to gluten-free bread significantly reduced lightness (L*), color saturation (C*), and hue angle (h°), while increasing total color difference (ΔE*). These trends are consistent with the present results where progressive enrichment with buckwheat husk led to darker crumb color, reduced chroma, perceptible hue shifts, and significantly higher ΔE* values, indicating notable visual changes compared to the control.

### 2.4. Evaluation of Sensory Properties

**Figure 1:**
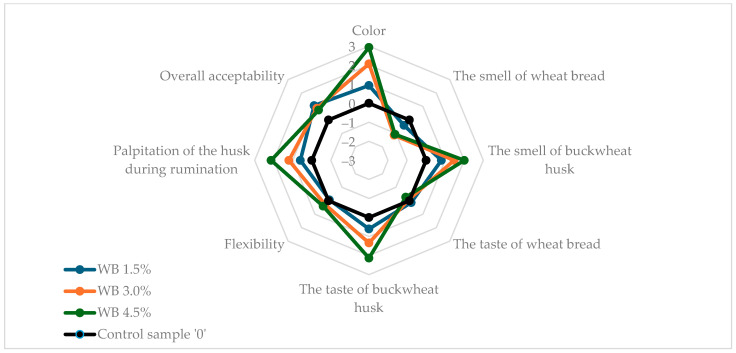
Sensory characteristics of analyzed wheat bread samples. WB stands for wheat bread with 0%, 1.5%, 3.0%, 4.5% addition of buckwheat husk.

**Figure 2:**
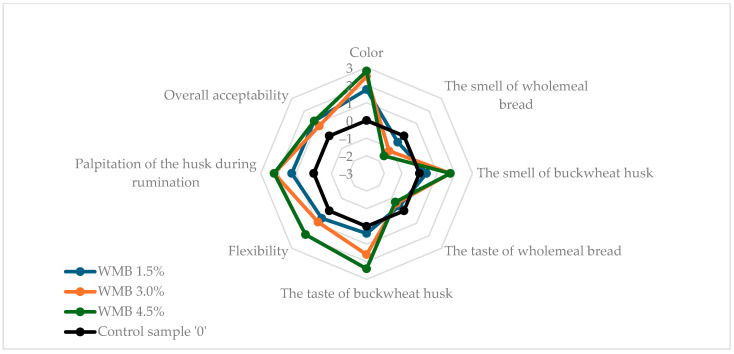
Sensory characteristics of analyzed wholemeal bread samples. WMB stands for wholemeal bread with 0%, 1.5%, 3.0%, 4.5% addition of buckwheat husk.

Figure 1 and Figure 2 show the results of the sensory evaluation of wheat and wholemeal breads enriched with buckwheat husk in comparison to control samples without husk addition. The buckwheat husk addition affected all tested sensory parameters in both wheat and wholemeal breads samples. In both types of bread, the addition of the husk reduced the intensity of the typical bread aroma even up to −1.6 in WMB 4.5%, but the characteristic bread flavor was retained. Apart from the WMB 1.5% bread, the smell and taste of the added husk were clearly noticeable. Compared to wheat bread, in wholemeal bread the addition of the husk clearly increased the flexibility of the bread, reaching the highest value (2.0) in the sample with the highest husk addition (WMB 4.5%). In wheat bread, the palpitation of the husk during chewing increased steadily at three levels of enrichment with husk (0.6, 1.2, 2.14 in WB 1.5%, WB 3.0% and WB 4.5%, respectively), while in wholemeal bread this attribute was scored for 1.25 in WMB 1.5% and 2.25 for both WMB 3.0% and WMB 4.5%. Similarly, with the color of wholemeal bread, the addition of the husk at 4.5% did not result in a visible increase in the color intensity of the bread compared to the 3.0% WMB (2.8) although the instrumental analysis showed a clear increase in the total color difference (ΔE*) between these two samples. This may be attributed to the already dark crumb color of wholemeal bread, which reduces the contrast perceived by observers; therefore, while instrumental methods can detect fine changes in color parameters, these differences may not always translate into noticeable sensory changes, especially when a visual saturation threshold is reached. Therefore, both methods provide complementary insights, and instrumental analysis confirms physical changes in crumb color, while sensory analysis reflects practical consumer perception. Conversely, in wheat bread, as the addition of the husk increased, the color of the bread became more intense (0.93, 2.07, 2.93 in WB 1.5%, WB 3.0%, and WB 4.5%, respectively). The results showed that wholemeal bread, due to its texture and darker color as a result of the addition of wholemeal flour, better masked the addition of husk at the highest enrichment level used in the experiment. Overall, acceptability of all tested bread samples with husk addition was rated higher than of control breads, reaching the highest value of 1.2 for WMB 1.5% and WMB 4.5%, which allowed the conclusion that the modified sensory characteristics of the bread through the addition of the husk would be attractive to consumers. The unexpectedly high acceptability of bread enriched with 4.5% husk may be attributed to the fine particle size of the powdered buckwheat husk, which contributed to the diversification of the structure and color of the crumb and gave it interesting flavor characteristics. Similar results were observed by Sakhare et al. (2013), who reported that bread made with finer fiber particles achieved better sensory qualities compared to those with coarser particles [49].

Similarly to Wronkowska et al. [50], who examined the addition of buckwheat husk to oat-based bread, we observed the potential of husk addition to modify and enhance some sensory attributes like flavor and color. On the contrary, we did not notice reduced flexibility of bread which indicates the importance of the raw material selection for husk enrichment in order to obtain the most desirable sensory characteristics of the final product. 

## 3. Materials and Methods

### 3.1. Bread Preparation

Two types of bread were prepared for this study. Wheat bread (WB 0) was formulated with 600 g wheat bread flour, corresponding to 60% of the total dough formulation (1000 g). Wholemeal bread (WMB 0) was prepared with 460 g wholemeal flour (46% of total formulation) and 240 g wheat bread flour (24% of total formulation). In all wholemeal formulations, the amount of wholemeal flour remained constant at 460 g (46% of total formulation), while the amount of wheat flour was reduced to 225 g (22.5%), 210 g (21.0%), and 195 g (19.5%) in WMB 1.5%, WMB 3.0%, and WMB 4.5%, respectively. Each bread type was enriched with grounded buckwheat husk at concentrations of 1.5%, 3.0%, and 4.5%. The tested breads ingredients are presented in Table 4. Ground buckwheat husk was bought from an online store in Poland, other ingredients were obtained from the local supermarket in Olsztyn (Northeast of Poland). The buckwheat husk used for fortification was obtained as a commercially available ground product (Bio Planet S.A., Leszno, Poland). According to the manufacturer, it is milled to a coarse powder; previous studies using similar products report that similar fine powders have a median particle size of approximately 62.7 µm [19]. The dough was mixed, leavened, and baked using an automated bread machine (Tefal PF6118, Is-sur-Tille, France) at a baking temperature of 200 °C. After the baking process was completed, the loaves were cooled to room temperature. The bread samples were processed using the vacuum blender (BOSCH VitaMaxx MMBV62M, Munich, Germany) to ensure thorough blending. This blender was employed to effectively homogenize the samples before further analysis.

### 3.2. Chemical and Reagents

Kits for water-soluble (ACW) and lipid-soluble antioxidants (ACL) for the photochemiluminesce (PCL) method were purchased from Analytik Jena (Jena, Germany), The total phenolic content (TPC) was determined using the Folin–Ciocalteu reagent (Aktyn, Suchy Las, Poland) and sodium carbonate (Stanlab, Lublin, Poland). For profiling total phenolic compounds, high-purity solvents and standard compounds were used. Methanol (MeOH), acetonitrile, water, formic acid (FA), and trifluoroacetic acid (TFA) of MS-grade purity were purchased from Merck (Darmstadt, Germany). A wide range of phenolic acid standards—including caffeic, chlorogenic, p-coumaric, ferulic, gentisic, hippuric, p-hydroxybenzoic, m-hydroxyphenylacetic, protocatechuic, salicylic, sinapic, syringic, and vanillic acids—along with flavonoid standards such as epicatechin, kaempferol, myricetin, and rutin, were obtained from Extrasynthese (Genay, Rodan, France) and Merck (Darmstadt, Germany). Gallic acid, used as a reference compound, was sourced from Sigma Aldrich (St. Louis, MO, USA). All reagents used were of analytical or MS-grade quality to ensure accuracy and reproducibility in phenolic compound profiling. Other chemicals used in the experiments were at least of analytical grade and were purchased from Merck (Darmstadt, Germany) and “POCH” S. A. (Gliwice, Poland).

### 3.3. Analysis of Antioxidant Activity

The photochemiluminesce (PCL) method described by Zieliński et al. [51] was used to determine the ability of bread samples to scavenge the superoxide anion radical (O_2_^•−^). Samples were diluted either with methanol in ACL or buffer in case of ACW. Analysis was carried out with the use of the Photochem^®^ apparatus (Analytik Jena, Jena, Germany). The results were calculated based on the Trolox standard curve (R^2^ = 0.9997 in ACW; R^2^ = 0.9999 in ACL) and presented as µmol Trolox eq./g of sample.

### 3.4. Determination of Total Content of Phenolic Compounds

The total content of phenolic compounds (TPC) in the analyzed bread samples was determined by a spectrophotometric method using the Folin–Ciocalteu reagent with gallic acid as a standard, according to Klepacka et al. [52]. Moreover, 0.5 mL of an appropriately prepared extract of phenolic compounds (which were first extracted three times with methanol and then concentrated and filtered into a 50 mL flask) was measured into a 10 mL volumetric flask and after, the Folin–Ciocalteu reagent and sodium carbonate were added. The samples were left in the dark for 1 h, and then the absorbance was measured at 765 nm against the blank samples (Thermo Scientific Helios Zeta UV-VIS, Madison, WI, USA). The samples were analyzed in triplicate. The results were expressed as gallic acid equivalent with a reference curve plotted for this acid:

y = 0.0223x − 0.1916, R^2^ = 0.990

### 3.5. Determination of Phenolic Compounds by HPLC

The profile and content of phenolic acids and flavonoids were assessed following the method described by Płatosz et al. [53]. Bread samples were first vortexed for 1 min, then sonicated for another 1 min using a VC 750 sonicator (Sonics & Materials Inc., Newtown, CT, USA). Afterward, the samples were centrifuged at 13,200× *g* for 20 min at 4 °C (5415 R Centrifuge, Eppendorf, Hamburg, Germany).

In the last step, samples were analyzed using an LC-MS system equipped with micro-HPLC (LC-200, Eksigent, Redwood, CA, USA) and a degasser, two binary pumps, and an autosampler. The LC-MS system was coupled with a QTRAP 5500 detector (AB Sciex, Vaughan, ON, Canada) consisting of a triple quadrupole, ion trap, and electrospray ionization (ESI) ion source. A total of 5 µL of pre-treated samples were injected into a HALO C18 column (0.5 mm × 100 mm × 2.7 μm, Eksigent, Vaughan, ON, Canada) kept at 45 °C. The flow rate was 15 μL/min with solvent A—water with 0.1% FA and solvent B—0.9% FA in a solution of acetonitrile with 9.1% methanol. The gradient started from 1% B for the first min. Next, the mobile phase composition was changed by increasing B to 90% (1 to 4 min) and returned to starting conditions in 0.5 min, keeping the re-equilibration at 1% B for 1 min. Data were collected in negative ion mode. The curtain gas was set to 20 L/min, and the following settings were used: collision gas: ion spray voltage: 5300 V; temperature: 350 °C; 1 ion source gas: 35 L/min; 2 ion source gas: 30 L/min; declastering potential: 100 V; entrance potential: 10 V; collision energy: 40 eV; and collision cell exit potential: 20 V. The identity and quantity of phenolic acids and flavonoids were confirmed by matching the experimental MS/MS spectra to MS/MS spectra from databases and fragmentation spectra and retention times (Multiple Reaction Monitoring, MRM) obtained for the standards. The linear calibration curves of external standards had correlation coefficients of 0.979–1.000. The results were expressed in µg/mL.

### 3.6. Color Analysis

Instrumental color measurements were performed on control bread samples and bread enriched with buckwheat husk using a CR-400 colorimeter (Konica Minolta Sensing Inc., Osaka, Japan) equipped with a D65 illuminant and the CIE 1931 standard 2° observer (color-matching functions derived from the Wright–Guild experiments that represent the average human response in a 2° visual field) [54]. All measurements were conducted in three places of the bread, i.e., one at the center and two on the side, at room temperature. The device was calibrated using a white ceramic plate provided by the manufacturer. The measurement results were expressed using the CIE Lab* color space, in which color is defined by the coordinates L* (light/dark), a* (red/green), and b* (yellow/blue).

Color measurements were performed in triplicate on the surface of both control and enriched bread samples to ensure accuracy and consistency. The color saturation (Chroma, C*), hue angle (h°), browning index (BI), and total color difference (ΔE*) were calculated using the following equations:

$$C^{*}=\sqrt{a^{*2}+b^{*2}}$$

h = arctan (b*/a*) + 180°

BI = 100(x − 0.31)/0.172

$$∆E^{*}=\sqrt{{∆L}^{*2}+{∆a}^{*2}+{∆b}^{*2}}$$

where

x = (a* + 1.75L*)/(5.645L* + a* − 0.012b*)

∆L* = lightness difference

∆a* = redness difference

∆b* = yellowness difference

### 3.7. Sensory Analysis

The tested wholemeal and wheat bread samples enriched with buckwheat husk were sensory evaluated using the differential profiling method by a trained team of ten panelist’s with an average age ranging from 22 to 40, with proven sensory sensitivity according to EN ISO 8586:2012 [55] from the Faculty of Food Sciences of the University of Warmia and Mazury in Olsztyn [56]. All bread samples, including the control, were blind-coded to minimize bias, and the order of presentation was randomized. Sensory analysis was carried out under controlled conditions in a sensory analysis laboratory. The panelist’s task was to mark on a bipolar scale how much a specific feature of the tested sample differs from the standards, which were not enriched with buckwheat husk test materials. A deviation scale from −3 (the lowest intensity of the tested sensory characteristic) to +3 (the highest intensity of the tested sensory characteristic), where 0 corresponded to the quality of the standard sample, was used. The scores for color, taste, odor, flexibility and overall acceptability were given by each member of the team. All procedures related to sensory evaluation were conducted in compliance with applicable laws and the ethical guidelines established by the Research Ethics Committee of the University of Warmia and Mazury in Olsztyn. Participation was entirely voluntary, with all individuals fully informed about the study’s purpose and scope. Participants provided informed consent, acknowledging that their responses would remain confidential. They also retained the right to withdraw from the study at any point without needing to provide a reason.

### 3.8. Statistical Analysis

All results were presented as the mean ± standard deviation of three replicates. The level of statistical significance among the means was analyzed by one-way ANOVA with Duncan’s post hoc test at a significance level of *p* ≤ 0.05 using Statistica software version 13.2 (StatSoft, Cracow, Poland).

## 4. Conclusions

This study increases the knowledge of possible uses of buckwheat husk to enhance the antioxidant potential of enriched bread. The improved antioxidant potential and enriched phenolic profile demonstrate that buckwheat husk can serve as a bioactive-rich ingredient supporting dietary strategies aimed at reducing oxidative stress and improving overall health. Although the enrichment led to visible darkening and textural changes, sensory evaluation revealed that overall acceptability was maintained or even improved, especially in wholemeal bread, which better masked the sensory impact of husk particles. However, increased perception of husk particles during chewing, especially at enrichment levels above 3% may result in a gritty mouthfeel (which depends not only on the amount of husk added, but also on the degree of its granulation). This must be taken into account when developing further consumer-acceptable formulations.

These findings highlight the dual benefit of utilizing buckwheat husk: valorizing an agricultural by-product and enhancing the functional profile of everyday foods. Moreover, the use of this natural, fiber-rich additive aligns with sustainable food development trends and offers an eco-friendly and health-promoting alternative to synthetic enhancers in the baking industry. The findings support further development of health-oriented cereal products enriched with bioactive-rich plant residues.

Future research should focus on improving the sensory quality of buckwheat husk enriched breads, optimizing particle size or pre-treatment methods, and investigating the bioavailability and health effects of buckwheat husk derived compounds.

## Figures and Tables

**Table 1:** Effect of buckwheat husk addition on antioxidant activity of tested bread samples.

Tested Samples	ACW (µmol Trolox/g dm)	Changes (%)	ACL (µmol Trolox/g dm)	Changes (%)	PCL (µmol Trolox/g dm)	Changes (%)	TPC (mg/g)	Changes (%)
WB 0	0.87 ± 0.02 ^c^		0.59 ± 0.02 ^d^		1.46 ± 0.00 ^d^		110.5 ± 6.3 ^b^	
WB 1.5%	0.79 ± 0.01 ^d^	−9.2%	1.39 ± 0.01 ^c^	135.5%	2.18 ± 0.00 ^c^	49.3%	96.3 ± 4.0 ^c^	−12.8%
WB 3.0%	1.20 ± 0.00 ^b^	37.9%	2.07 ± 0.03 ^b^	251.6%	3.26 ± 0.03 ^b^	123.3%	128.8 ± 3.9 ^a^	16.5%
WB 4.5%	1.29 ± 0.01 ^a^	48.3%	2.73 ± 0.04 ^a^	362.7%	4.01 ± 0.03 ^a^	174.7%	91.0 ± 5.8 ^c^	−17.6%
WMB 0	1.40 ± 0.04 ^c^		2.43 ± 0.03 ^d^		3.84 ± 0.07 ^d^		97.0 ± 6.1 ^c^	
WMB 1.5%	1.52 ± 0.02 ^b^	8.5%	2.84 ± 0.01 ^c^	16.8%	4.36 ± 0.01 ^c^	13.5%	111.8 ± 4.3 ^b^	15.2%
WMB 3.0%	1.58 ± 0.03 ^b^	12.8%	3.38 ± 0.12 ^b^	39.0%	4.95 ± 0.09 ^b^	28.9%	127.8 ± 5.6 ^a^	31.7%
WMB 4.5%	1.71 ± 0.03 ^a^	22.1%	3.91 ± 0.06 ^a^	60.9%	5.62 ± 0.03 ^a^	46.3%	131.2 ± 3.1 ^a^	35.2%

Values are expressed as means (*n* = 3) ± standard deviations; dm–dry mass. ACW—antioxidant compounds soluble in water; ACL—antioxidant compounds soluble in lipids; PCL is the sum of results for ACW and ACL; TPC is the total phenolic content. Changes (%) were calculated relative to their respective control samples using the formula: Changes% = [(ACL sample − ACL control)/ACL control] × 100; the values (−) minus means decrease when compared to control sample. WB 0—wheat bread without buckwheat husk; WB 1.5%, WB 3.0%, and WB 4.5%—wheat breads with 1.5%, 3.0%, and 4.5% buckwheat husk addition, respectively; WMB 0—wholemeal bread without buckwheat husk; WMB 1.5%, WMB 3.0%, and WMB 4.5%—wholemeal breads with 1.5%, 3.0%, and 4.5% buckwheat husk addition, respectively. Values in the same column marked with different lowercase letters (a–d) are significantly different at *p* ≤ 0.05 according to Duncan’s test.

**Table 2:** Content of polyphenols (phenolic acids and flavonoids).

	Wheat Bread				Wholemeal Bread			
Addition of Buckwheat Husk	WB 0%	WB 1.5%	WB 3.0%	WB 4.5%	WMB 0%	WMB 1.5%	WMB 3.0%	WMB 4.5%
phenolic acids (µg/g)								
p-hydroxybenzoic acid	0.018 ± 0.003 ^b^	0.034 ± 0.003 ^a^	0.006 ± 0.001 ^c^	0.003 ± 0.000 ^c^	0.004 ± 0.000 ^a^	0.003 ± 0.001 ^a^	0.005 ± 0.001 ^a^	0.004 ± 0.001 ^a^
salicylic acid	0.002 ± 0.000 ^c^	0.002 ± 0.000 ^c^	0.004 ± 0.001 ^b^	0.007 ± 0.001 ^a^	0.003 ± 0.001 ^b^	0.003 ± 0.000 ^b^	0.005 ± 0.001 ^b^	0.053 ± 0.004 ^a^
protocatechuic acid	0.007 ± 0.002 ^c^	0.129 ± 0.017 ^b^	0.349 ± 0.039 ^a^	0.370 ± 0.070 ^a^	0.020 ± 0.003 ^a^	0.018 ± 0.003 ^a,b^	0.014 ± 0.000 ^b^	0.018 ± 0.002 ^b^
gentisic acid	0.016 ± 0.002 ^b^	0.015 ± 0.002 ^b^	0.025 ± 0.004 ^a^	0.019 ± 0.003 ^a,b^	0.053 ± 0.009 ^b^	0.116 ± 0.016 ^a^	0.053 ± 0.005 ^b^	0.053 ± 0.002 ^b^
p-coumaric acid	0.218 ± 0.029 ^b^	0.231 ± 0.040 ^b^	0.406 ± 0.016 ^a^	0.472 ± 0.052 ^a^	0.109 ± 0.022 ^b^	0.115 ± 0.015 ^b^	0.086 ± 0.014 ^b^	0.228 ± 0.024 ^a^
o-coumaric acid	0.002 ± 0.000 ^a^	0.002 ± 0.000 ^a^	0.002 ± 0.000 ^a^	0.002 ± 0.000 ^a^	0.001 ± 0.000 ^b^	<0.001	0.001 ± 0.000 ^a,b^	0.001 ± 0.000 ^a^
vanilic acid	0.112 ± 0.021 ^b^	0.204 ± 0.033 ^b^	0.518 ± 0.038 ^a^	0.522 ± 0.057 ^a^	0.234 ± 0.010 ^c^	0.271 ± 0.045 ^c^	0.739 ± 0.109 ^b^	1.045 ± 0.162 ^a^
hippuric acid	0.002 ± 0.000 ^c^	0.007 ± 0.001 ^b^	0.005 ± 0.001 ^b,c^	0.017 ± 0.002 ^a^	0.013 ± 0.001 ^b^	0.007 ± 0.001 ^b^	0.025 ± 0.006 ^a^	0.012 ± 0.002 ^b^
caffeic acid	0.007 ± 0.001 ^b^	0.014 ± 0.003 ^b^	0.011 ± 0.001 ^b^	0.061 ± 0.010 ^a^	0.032 ± 0.003 ^c^	0.008 ± 0.001 ^c^	0.076 ± 0.012 ^b^	0.166 ± 0.023 ^a^
ferulic acid	2.170 ± 0.269 ^b^	0.532 ± 0.071 ^c^	2.816 ± 0.295 ^a^	1.782 ± 0.248 ^b^	4.117 ± 0.323 ^b^	5.981 ± 0.987 ^a^	0.613 ± 0.116 ^c^	0.300 ± 0.050 ^c^
syringic acid	1.465 ± 0.282 ^b^	1.600 ± 0.305 ^b^	2.306 ± 0.331 ^b^	3.213 ± 0.376 ^a^	2.106 ± 0.361 ^b^	2.534 ± 0.349 ^b^	2.084 ± 0.196 ^b^	4.275 ± 0.570 ^a^
chlorogenic acid	<0.001	0.001 ± 0.000 ^c^	0.003 ± 0.000 ^b^	0.004 ± 0.001 ^a^	<0.001	<0.001	<0.001	<0.001
sinapic acid	0.101 ± 0.017 ^a^	0.074 ± 0.009 ^a^	0.079 ± 0.010 ^a^	0.026 ± 0.005 ^b^	0.426 ± 0.045 ^b^	0.610 ± 0.062 ^a^	0.626 ± 0.095 ^a^	0.490 ± 0.047 ^a,b^
Sum of phenolic acids	4.12 ± 0.39 ^b^	2.84 ± 0.32 ^c^	6.53 ± 0.45 ^a^	6.50 ± 0.46 ^a^	7.12 ± 0.49 ^b^	9.67 ± 1.05 ^a^	4.33 ± 0.27 ^d^	6.64 ± 0.6 ^c^
flavonoids (µg/g)								
apigenin	0.001 ± 0.000	0.001 ± 0.000	0.001 ± 0.000	0.001 ± 0.000	<0.001	<0.001	0.001 ± 0.000	0.001 ± 0.000
naringenin	<0.001	<0.001	<0.001	<0.001	<0.001	<0.001	<0.001	<0.001
kaempferol	0.002 ± 0.000	0.002 ± 0.000	0.002 ± 0.000	0.002 ± 0.000	nd	nd	nd	nd
luteolin	<0.001	<0.001	<0.001	<0.001	<0.001	<0.001	<0.001	<0.001
catechin	0.006 ± 0.001 ^c^	0.015 ± 0.003 ^b^	0.021 ± 0.004 ^b^	0.044 ± 0.005 ^a^	0.008 ± 0.001 ^b^	0.019 ± 0.003 ^a^	0.006 ± 0.001 ^b^	0.005 ± 0.001 ^b^
quercetin	<0.001	<0.001	<0.001	<0.001	<0.001	<0.001	<0.001	<0.001
isorhamnetin	<0.001	<0.001	0.001 ± 0.000	0.001 ± 0.000	<0.001	<0.001	<0.001	0.005 ± 0.001
myricetin	0.001 ± 0.000 ^c^	0.001 ± 0.000 ^c^	0.016 ± 0.002 ^b^	0.030 ± 0.003 ^a^	0.001 ± 0.000 ^c^	0.002 ± 0.001 ^a^	0.002 ± 0.000 ^b^	0.004 ± 0.000 ^a^
orienthin	0.009 ± 0.001 ^c^	0.036 ± 0.003 ^c^	0.118 ± 0.012 ^b^	0.247 ± 0.039 ^a^	0.007 ± 0.001 ^c^	0.036 ± 0.006 ^b,c^	0.081 ± 0.006 ^b^	0.354 ± 0.034 ^a^
rutin	nd	0.001 ± 0.000 ^c^	0.002 ± 0.000 ^b^	0.003 ± 0.000 ^a^	nd	<0.001	0.001 ± 0.000	0.001 ± 0.000
vitexin	0.002 ± 0.000 ^b,c^	0.001 ± 0.000 ^c^	0.002 ± 0.000 ^b^	0.005 ± 0.001 ^a^	0.002 ± 0.000 ^b^	0.002 ± 0.000 ^b^	0.003 ± 0.001 ^b^	0.024 ± 0.005 ^a^
Sum of flavonoids	0.02 ± 0.0 ^d^	0.06 ± 0.0 ^c^	0.16 ± 0.01 ^b^	0.33 ± 0.04 ^a^	0.02 ± 0.0 ^d^	0.06 ± 0.01 ^c^	0.09 ± 0.01 ^b^	0.39 ± 0.03 ^a^
Sum of phenolic acids and flavonoids (µg/g)	4.14 ± 0.39 ^b^	2.9 ± 0.32 ^c^	6.69 ± 0.5 ^a^	6.83 ± 0.46 ^a^	7.14 ± 0.49 ^b^	9.73 ± 1.05 ^a^	4.42 ± 0.27 ^c^	7.03 ± 0.6 ^b^

Values are expressed as means (*n* = 3) ± standard deviations. Mean values for phenolic acids and flavonoids in wheat and wholemeal breads with varying buckwheat husk additions (0, 1.5%, 3.0%, and 4.5%) are presented. WB 0—Wheat bread without buckwheat husk; WB 1.5%, WB 3.0%, and WB 4.5%—wheat breads with 1.5%, 3.0%, and 4.5% buckwheat husk addition, respectively; WMB 0-wholemeal bread without buckwheat husk; WMB 1.5%, WMB 3.0%, and WMB 4.5%—wholemeal breads with 1.5%, 3.0%, and 4.5% buckwheat husk addition, respectively. Mean values for breads before and after the addition of buckwheat husk with different lowercase letters in the row are statistically different (*p* ≤ 0.05) according to the Duncan multiple range test; nd—not detected. Values in the same rows marked with different lowercase letters (a–d) are significantly different at *p* ≤ 0.05 according to Duncan’s test.

**Table 3:** Browning index and CIE lab-based color metrics of wheat and wholemeal breads enriched with buckwheat husk.

Samples	Browning Index (BI)	Lightness (L*)	Color Saturation (C*)	Hue Angle (h°)	Total Color Difference (ΔE*)
WB 0	2.98 ± 0.24 ^c^	75.31 ± 2.24 ^a^	14.43 ± 0.81 ^a^	77.62 ± 0.64 ^a^	n.d.
WB 1.5%	3.32 ± 0.43 ^c^	67.95 ± 3.30 ^b^	10.62 ± 0.27 ^b^	72.78 ± 2.57 ^b^	8.43 ± 3.04 ^b^
WB 3.0%	4.24 ± 0.16 ^b^	62.65 ± 1.72 ^c^	9.59 ± 0.84 ^b^	67.04 ± 1.78 ^c^	13.73 ± 1.92 ^b^
WB 4.5%	5.26 ± 0.14 ^a^	59.00 ± 0.40 ^c^	10.26 ± 0.54 ^b^	64.78 ± 0.76 ^c^	17.05 ± 0.49 ^a^
WMB 0	5.74 ± 0.11 ^b^	73.81 ± 1.34 ^a^	14.53 ± 0.70 ^a^	65.72 ± 0.84 ^a,b^	n.d.
WMB 1.5%	4.81 ± 0.53 ^b^	66.67 ± 1.57 ^b^	10.98 ± 1.56 ^b^	65.73 ± 0.27 ^a,b^	7.40 ± 2.12 ^c^
WMB 3.0%	5.64 ± 0.33 ^b^	59.60 ± 0.64 ^c^	10.71 ± 0.62 ^b^	63.72 ± 0.33 ^b^	14.08 ± 0.45 ^b^
WMB 4.5%	6.78 ± 0.80 ^a^	54.32 ± 0.91 ^d^	12.99 ± 1.62 ^a,b^	66.32 ± 1.92 ^a^	18.94 ± 0.75 ^a^

WB 0—wheat bread without buckwheat husk; WB 1.5%, WB 3.0%, and WB 4.5%—wheat breads with 1.5%, 3.0%, and 4.5% WMB 0—wholemeal bread without buckwheat husk; WMB 1.5%, WMB 3.0%, and WMB 4.5%—wholemeal breads with 1.5%, 3.0%, and 4.5% buckwheat husk addition, respectively. Mean values for breads before and after the addition of buckwheat husk with different lowercase letters in the columns are statistically different (*p* ≤ 0.05) according to the Duncan multiple range test; n.d.—not detected. Values in the same column marked with different lowercase letters (a–d) are significantly different at *p* ≤ 0.05 according to Duncan’s test.

**Table 4:** Tested breads formulae.

Type of Bread	Ingredients [g/1000 g]
WB 0	Wheat bread flour type 650 (600), baker’s yeast (1.5), salt (8), oil (9), sugar (10), milk powder (10), water (361.5)
WB 1.5%	Wheat bread flour type 650 (585), buckwheat husk (15), baker’s yeast (1.5), salt (8), oil (9), sugar (10), milk powder (10), water (361.5)
WB 3.0%	Wheat bread flour type 650 (570), buckwheat husk (30), baker’s yeast (1.5), salt (8), oil (9), sugar (10), milk powder (10), water (361.5)
WB 4.5%	Wheat bread flour type 650 (555), buckwheat husk (45), baker’s yeast (1.5), salt (8), oil (9), sugar (10), milk powder (10), water (361.5)
WMB 0	Wheat bread flour type 650 (240), wholemeal wheat flour type 2000 (460), baker’s yeast (1.5), salt (8), oil (9), sugar (10), milk powder (10), water (261.5)
WMB 1.5%	Wheat bread flour type 650 (225), wholemeal wheat flour type 2000 (460), buckwheat husk (15), baker’s yeast (1.5), salt (8), oil (9), sugar (10), milk powder (10), water (261.5)
WMB 3.0%	Wheat bread flour type 650 (210), wholemeal wheat flour type 2000 (460), buckwheat husk (30), baker’s yeast (1.5), salt (8), oil (9), sugar (10), milk powder (10), water (261.5)
WMB 4.5%	Wheat bread flour type 650 (195), wholemeal wheat flour type 2000 (460), buckwheat husk (45), baker’s yeast (1.5), salt (8), oil (9), sugar (10), milk powder (10), water (261.5)

WB 0—wheat bread without buckwheat husk; WB 1.5%, WB 3.0%, and WB 4.5%—wheat breads with 1.5%, 3.0%, and 4.5% buckwheat husk addition, respectively; WMB 0—wholemeal bread without buckwheat husk; WMB 1.5%, WMB 3.0%, and WMB 4.5%—wholemeal breads with 1.5%, 3.0%, and 4.5% buckwheat husk addition, respectively. Water was adjusted as needed to achieve proper dough consistency.

## Data Availability

The original contributions presented in this study are included in the article. Further inquiries can be directed to the corresponding author.
